# System dynamics modeling for traumatic brain injury: Mini-review of applications

**DOI:** 10.3389/fbioe.2022.854358

**Published:** 2022-08-12

**Authors:** Erin S. Kenzie, Elle L. Parks, Nancy Carney, Wayne Wakeland

**Affiliations:** ^1^ Oregon Rural Practice-Based Research Network, Oregon Health and Science University, Portland, OR, United States; ^2^ Systems Science Program, Portland State University, Portland, OR, United States; ^3^ Independent Researcher, Portland, OR, United States; ^4^ Department of Medical Informatics and Clinical Epidemiology, Oregon Health and Science University, Portland, OR, United States

**Keywords:** traumatic brain injury, system dynamics, modeling, systems science, complexity, simulation

## Abstract

Traumatic brain injury (TBI) is a highly complex phenomenon involving a cascade of disruptions across biomechanical, neurochemical, neurological, cognitive, emotional, and social systems. Researchers and clinicians urgently need a rigorous conceptualization of brain injury that encompasses nonlinear and mutually causal relations among the factors involved, as well as sources of individual variation in recovery trajectories. System dynamics, an approach from systems science, has been used for decades in fields such as management and ecology to model nonlinear feedback dynamics in complex systems. In this mini-review, we summarize some recent uses of this approach to better understand acute injury mechanisms, recovery dynamics, and care delivery for TBI. We conclude that diagram-based approaches like causal-loop diagramming have the potential to support the development of a shared paradigm of TBI that incorporates social support aspects of recovery. When developed using adequate data from large-scale studies, simulation modeling presents opportunities for improving individualized treatment and care delivery.

## Introduction

Worldwide, sixty-nine million people sustain traumatic brain injury (TBI) annually (Dewan et al., 2018). Experiencing TBI can have long-term health consequences that affect not only the patient, but also caregivers, extended families, and communities (Eunice Kennedy Shriver National Institute of Child Health and Human Development, 1993; Hyder et al., 2007; James et al., 2019; Malec et al., 2017; Qadeer et al., 2017; Iaccarino et al., 2018; Carlozzi et al., 2020; Sodders et al., 2020). Patients with TBI often experience physical, behavioral, emotional, and cognitive difficulties that can persist many years post-injury and affect crucial aspects of everyday life, including independence, mobility, employment, and community integration (Finnanger et al., 2013; Arango-Lasprilla et al., 2018; Seagly et al., 2018). Social and interpersonal impacts have been shown to significantly interfere with community living, occupational status, and sustainment of interpersonal relationships (Marsh and Knight 1991; Franulic et al., 2000). For caregivers of patients with TBI, compromised physical and mental health, social participation, family functioning, social and spousal relationships, employment, finances, and self-care have been well documented (Saban et al., 2016; Griffin et al., 2017; Brickell et al., 2019; Dreer et al., 2019).

TBI has been identified as the “most complex disease in the most complex organ” of the body, (Wheble and Menon 2016), spanning biomechanical, neurochemical, neurological, cognitive, emotional, and social dimensions (Kenzie et al., 2017; 2018). Despite the establishment of evidence-based treatment guidelines (Carney et al., 2017) and considerable investment in large, multi-centered research consortia (Yue et al., 2013; Cifu et al., 2015; Ivory and Bellgowan 2015; Maas et al., 2015), little progress has been made in acute care therapeutics for TBI, and clinical trials addressing acute care consistently fail (Narayan 2002; Samadani 2016). Similarly, rehabilitation for TBI has shown minimal progress. A 1999 systematic review of cognitive rehabilitation for TBI reported insufficient evidence for the effectiveness of this standard intervention (Carney et al., 1999). Twelve years later, the Institute of Medicine reported the same findings (IOM 2011).

Researchers and clinicians urgently need a rigorous conceptualization of brain injury that encompasses nonlinear and mutually causal relations among the factors involved, as well as sources of individual variation in recovery trajectories. The fundamental necessity of this type of systems approach has been advocated by medical and scientific research experts for nearly 30 years. The National Center for Medical Rehabilitation Research (NCMRR) published a research plan that rejected the traditional linear view of rehabilitation and encouraged the adoption of a systems approach as “an essential feature of medical rehabilitation research and, ultimately, all health care delivery” (1993). Unfortunately, this mandate has not been accomplished, and recent TBI research continues the call for a systems perspective (Bigler 2016; Kenzie et al., 2017; Kenzie et al., 2018).

System dynamics, an approach from complex systems science (Sterman 2000), involves modeling nonlinear feedback relationships that produce nonlinear behavior, either with diagrams or simulations based on ordinary differential equations (ODEs). This approach has been used in several ways to address the complexity of TBI and provide tools to facilitate understanding and aid diagnostic and prognostic capabilities. The purpose of this mini-review is to summarize and compare current applications of system dynamics modeling for TBI and highlight opportunities for further development.

## Methods

To locate studies using system dynamics modeling for TBI research, we searched Google Scholar and PubMed using relevant terms. We focused on articles published in the past 15 years that used system dynamics modeling (e.g., causal-loop diagramming or system dynamics simulation) in some aspect of TBI pathophysiology, diagnosis, prognosis, treatment, recovery, or care delivery. Because much of this research is emerging, we included conference publications. Articles were not vetted for quality due to the small number of studies identified and the descriptive nature of this brief review.

## Results

Ten publications were identified that fit the search criteria. Included studies span a variety of fields, approaches, and research questions. Table 1 summarizes the purpose of modeling and the approach used for each study.

**TABLE 1:** Applications of system dynamics modeling for traumatic brain injury.

Publication	Purpose of modeling	Approach
Acute injury mechanisms		
Kochis et al. (2021)	To identify time course of potential TBI biomarkers	Computational system dynamics model of GFAP and IgG in bloodstream over time following TBI
Scheff et al. (2013)	To model inflammatory response to trauma	Conceptual multi-scale ODE model of inflammatory response to trauma, including data-driven model of endotoxemia, transcriptional processes and cellular signaling cascades, modeling of immunomodulatory hormones and influence of cortisol and epinephrine on heart rate variability
Vaughan et al. (2018)	To model interactions between pro- and anti-inflammatory cytokines, microglia, and CNS tissue damage over time in severe TBI	Complex set of ODEs calibrated to patient data collected in the first 5 days post injury for 3 subgroups; parameter differences by group used to identify mechanistic differences in their neuroinflammatory patterns and outcomes
Wakeland et al. (2009)	To test prediction potential of model that calculates ICP following TBI	ODE model of blood volume and pressure within the brain, as influenced by hematoma, brain swelling and CSF
Complex recovery dynamics		
Kenzie et al. (2018)	To describe feedback dynamics across cellular, network, experiential, and social levels of mTBI recovery	Causal-loop diagram developed through literature review, group modeling sessions, and individual interviews with TBI experts
Wakeland and Kenzie (2019)	To describe estimated TBI recovery trajectories based on variable individual inputs	Proof-of-concept simulation model based on causal-loop diagram; many approximations included
Care delivery		
Jones et al. (2012)	To examine the potential impacts of implementing a performance improvement target (reduced emergency department wait times) in a hospital system; time to CT for TBI is secondary outcome measure	Computational system dynamics model describing patient flow in an emergency department; model developed in collaboration with stakeholder panel
Lee et al. (2015)	To estimate the current and future prevalence of people with intellectual developmental disabilities (e.g., from TBI) in New South Wales	Computational system dynamics model based on administrative data; modeler-led
Pilar et al. (2020)	To improve strategies for implementing TBI clinical care guidelines in clinical practice; focused on improving consistency of care and guideline-informed decision-making in three pediatric ICUs treating severe TBI in children	Causal-loop diagrams of ICU workflow developed through group modeling sessions with clinical care stakeholders; diagrams were then integrated with a novel technology-based engine for clinical decision-making that implemented evidence-based best practices at key points in care delivery
Vogel et al. (2019)	To support improved implementation of evidence-based guidelines for pediatric severe TBI in the ICU, determine similarities and differences across ICU cultures and provider types, and to identify structural features and crucial leverage points in ICU systems	Causal-loop diagram developed through group modeling sessions with three groups of stakeholders (nurses, trainees, and attending physicians) at each of three study sites; resulting diagram will be used to develop a novel technology-based intervention to support evidence based decision-making for ICU patients with TBI

CSF, cerebrospinal fluid; CT, computerized tomography; GFAP, glial fibrillary acidic protein; ICU, intensive care unit; IgG, immunoglobulin G; ODE, ordinary differential equation; mTBI, mild traumatic brain injury; TBI, traumatic brain injury.

### Acute injury mechanisms

Some studies used system dynamics to model specific pathophysiological mechanisms, such as intracranial pressure (ICP) and inflammation. Because system dynamics modeling involves accumulations and flows, it is especially well suited for modeling ICP. Although the properties of brain regions can differ significantly, treating volumes and pressures in an aggregate fashion is appropriate due to the rigid volume constraint of the intracranial cavity.

Early researchers used electrical circuit analogs and their associated ODEs to study elevated ICP resulting from hematoma and brain swelling during the acute post-injury phase of TBI (Marmarou 1973 and Ursino 1988). The mathematics of system dynamics is identical to these electrical analogs and has the advantage of a diagrammatic visualization that is more comprehensible for most clinicians than electrical circuit diagrams. Wakeland and Goldstein (2008) for a review of computational ICP models. Early pioneers in the computational modeling of ICP dynamics were able to match empirical ICP trajectory data remarkably well, aided by their detailed logic for cerebral autoregulation (Marmarou et al., 1978; Ursino and Alberto Lodi 1997). Models developed by Ursino and his team included multiple aspects of cerebral autoregulation, calibrated with data from a prospective study in which patients were given mild ventilation challenges (Ursino et al., 2000), and showed excellent prediction potential. Wakeland et al. (2009) also calibrated a systems dynamics ICP model on patient data collected prospectively for pediatric TBI patients. In this model, patients were given mild physiologic challenges (raising or lowering the head of the bed or moderately adjusting ventilator settings) at multiple time points, but results revealed inadequate clinical prediction capability due to patient responses to a dramatically varying stimulus. Clinical usage of computer models to improve the treatment of pediatric patients will be limited until these differential responses seen clinically are better understood. Moreover, using an aggregate modeling method to understand the etiology of local regions of ischemia would likely be very difficult and would require instead a more spatially explicit methodology. In some cases, a hybrid methodology with some aspects being treated as aggregate quantities and others being highly localized might be effective. Disaggregated aspects could be modeled using finite elements or agent-oriented logic.

Scheff et al. (2013) present an intriguing differential equation-based model involving inflammatory responses to injury at multiple scales. Researchers first developed a data-driven physio-chemical network model of endotoxemia focused on transcriptional processes and cellular signaling cascades. Next, an indirect response model of the pharmacokinetics and dynamics of immunomodulatory was described. These molecular-level cascades influence cortisol and epinephrine and therefore heart rate variability, which previous research indicates is correlated with disease state. However, the model was not calibrated with or verified against patient data.

Vaughan et al. (2018) developed a differential equation model of the interactions between pro-and anti-inflammatory cytokines (IL-1B, IL-4, IL-10, IL-12), M1-and M2-like microglia, and central nervous system tissue damage. The model replicated the complex cascades associated with neuroinflammation and was calibrated using data collected during the first 5 days following severe TBI using the Matlab toolset. Patients were classified into three subgroups, and the optimal model parameter values by subgroup revealed distinct mechanistic differences in neuroinflammatory patterns. The resulting increase in understanding of microglia pathophysiology will help to improve acute TBI treatment.

Kochis et al. (2021) developed a simulation model describing the concentrations of glial fibrillary acidic protein (GFAP) and immunoglobulin G (IgG) in the bloodstream following TBI with and without repeated injury. Understanding the rates of change of these levels throughout recovery has the potential of informing the use of these biomarkers for clinical assessment of injury severity and recovery.

### Complex recovery dynamics

**FIGURE 1:**
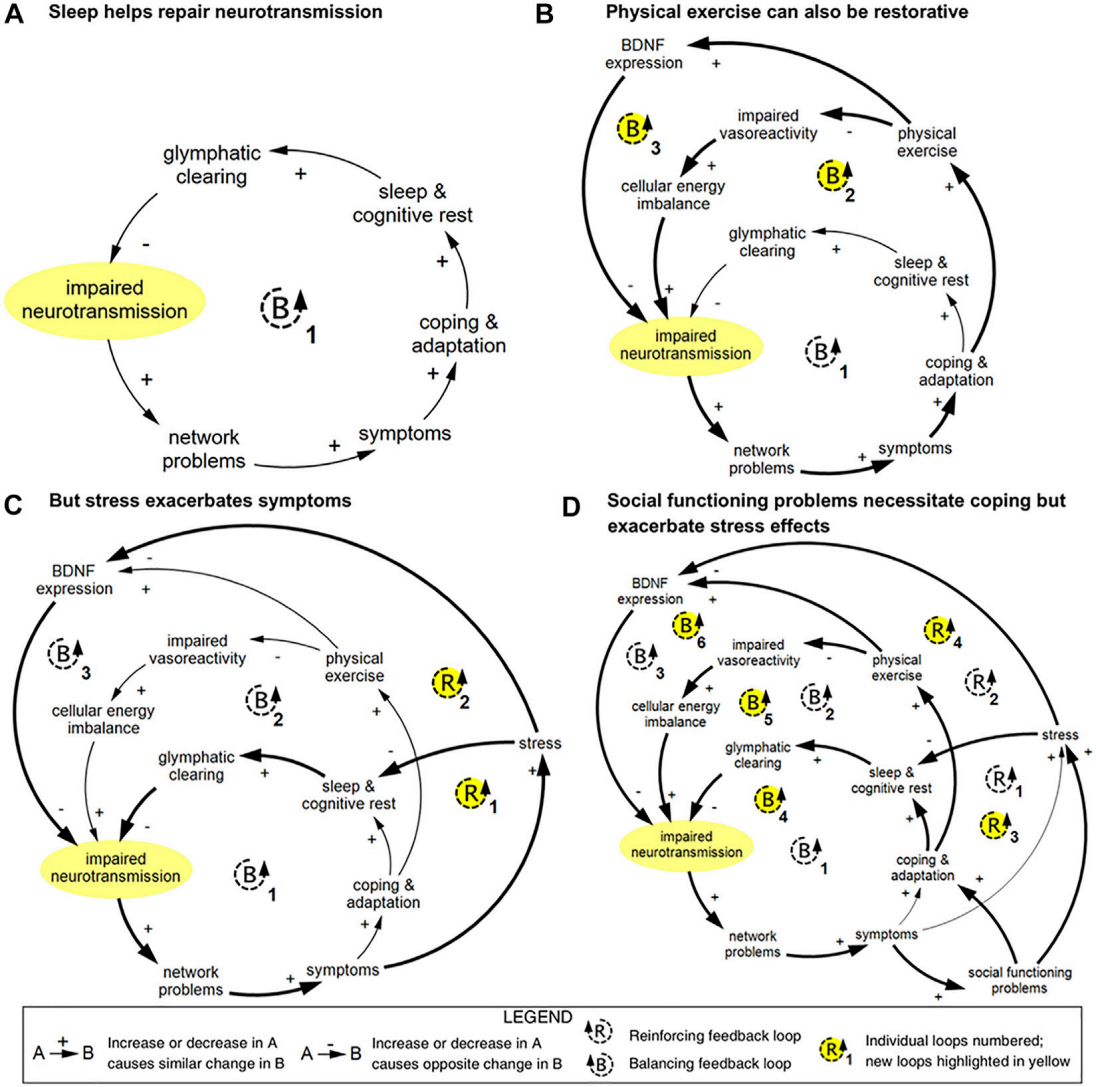
Example of causal-loop diagram showing feedback loops pertaining to impaired neurotransmission in TBI, reproduced from Kenzie et al. (2018). This series of diagrams illustrates how connected loops can have compounding and counteractive effects. **(A)** In loop B1, impaired neurotransmission affects the function of networks; these networks and network functions include limbic, intrinsic connectivity networks, attentional filtering, and processing speed. Disruption in these networks results in a range of symptoms that prompt coping and adaptation strategies. Restorative sleep processes lead to glymphatic clearing of brain waste and energy byproducts, which in turn results in improved neurotransmission *via* an improved cellular milieu and support of neuroplasticity. **(B)** In loop B2, physical exercise is used as a coping and adaptation strategy, which improves vasoreactivity and cellular energy imbalance, which supports neurotransmission. In loop B3, brain-derived neutrophic factor (BDNF) expression is strengthened, which reduces impaired neurotransmission *via* improved neuroplasticity. **(C)** Stress can disrupt sleep and inhibit BDNF expression, which creates two reinforcing loops. **(D)** Social functioning problems can prompt coping and adaptation, which introduces three additional balancing loops, and increase stress, which compounds the reinforcing effects of stress. Diagrams rendered in MapSys.

To describe the complex dynamics of recovery across multiple levels, members of the current author team previously developed a conceptual systems framework for studying concussion (Kenzie et al., 2017), followed by a causal-loop diagram of mTBI recovery (Kenzie et al., 2018) and preliminary computational system dynamics model of mTBI recovery patterns (Wakeland and Kenzie 2019). The causal-loop diagram and subdiagrams, one of which is shown in Figure 1, visually illustrate interactions between variables at cellular, network, experiential, and social levels, particularly feedback relationships that form the basis of nonlinear system behavior. It was created through an iterative process involving literature review and consultation with subject matter experts. It serves as a synthesis of the current scientific understanding of recovery dynamics in mTBI.

A computational model based upon the basic causal structure of the causal-loop diagram discussed above was able to generate estimated recovery trajectories for all severities of TBI given different inputs (Wakeland and Kenzie 2019). Due to limitations in obtaining high-quality time course data to inform the simulation model, many of the equations included in the model were approximations. The model therefore serves as an illustration of the potential capabilities of computational system dynamics to model TBI recovery trajectories.

### Care delivery

Even when evidence-based guidelines for TBI exist, implementation of guidelines in clinical practice remains limited and challenging (Pilar et al., 2020). Yet systems dynamics approaches can also be used to create specific guideline-based care strategies for medical teams and hospitals in complex fast-paced, transdisciplinary working environments. Pilar et al. (2020) used systems dynamics group model building to elicit stakeholder knowledge about three regional pediatric intensive care units (ICUs) treating children with severe TBI. Three groups of stakeholders (nurses, trainees, and attending physicians) were engaged in nine group model building sessions focused on factors related to timely decision-making and guideline-based workflows. Gathering information in this way from multiple stakeholders revealed crucial dynamics consistent across each ICU hospital site regarding communication, standardized protocols, TBI-related knowledge and education of the clinical provider(s), resources, and leadership. These dynamics were captured in causal-loop diagrams of ICU workflow that were then integrated with a novel clinical implementation strategy—a computer technology-based “bedside guideline engine” to facilitate timely and consistent delivery of evidence-based TBI pediatric care (Pilar et al., 2020). Similar mixed methods systems have been utilized to improve emergency department care and analyze how systems dynamics in a single department affect broader hospital performance, patient care, and outcomes over time (Jones et al., 2012).

Dynamic systems models of patient and clinical workflows over time thus provide strategies that may be singularly suited for implementing care guidelines consistently in multiple complex medical environments. Systems modeling techniques have also demonstrated utility for predicting what resources for clinical care may be needed in the future. For example, Lee et al. (2015) identified from government data the number of individuals in New South Wales whose characteristics met the criteria for ‘intellectual developmental disorders’ (including TBI) and then developed a computational systems dynamics model to predict the prevalence of these disorders in 2043, with the goal of more effectively preparing for future healthcare needs.

## Discussion

To address the need for complexity-informed approaches for understanding TBI injury and recovery, system dynamics has been used in recent years to model isolated mechanisms, complex relationships between factors in recovery, and aspects of care delivery. Approaches range from highly speculative mathematical models to diagram-based and group modeling methods. The scope of these models also varies widely, from specific mechanisms to a comprehensive understanding of the broad spectrum of processes involved in recovery. These differences reflect the range of capabilities of system dynamics modeling more broadly (Sterman 2000).

A defining characteristic of system dynamics modeling is that it is mechanistic, meaning that it involves *a priori* determination of hypothesized causal relationships between variables in a system. This determination can be closely tied to prior scientific literature or other sources (Sterman 2000; Kenzie 2021) co-created through a group process (Andersen et al., 1997; Vennix 1999; Andersen et al., 2007; Luna-Reyes et al., 2018), or made by an individual modeler (Sterman 2000), depending on the aims of the modeling effort. Contrasted with data-driven modeling approaches that treat causal processes generating system behavior as a “black box,” system dynamics modeling requires direct consideration of causal processes and mechanisms, which can support the synthesis of current scientific knowledge about a phenomenon, and can generate new hypotheses, and shared understanding within a field (Ghaffarzadegan et al., 2011; Aerts et al., 2014; Naumann et al., 2019). System dynamics simulation models can be used for *in silico* experimentation to preliminarily test hypotheses when direct experimentation is impractical or unethical (Lombardo et al., 2021). These approaches, particularly diagram-based methods like causal-loop diagramming, allow for the integration of heterogeneous types of variables, such as the interactions between social support and biophysical mechanisms of recovery. Moreover, information can come from different sources with varying degrees of evidence support, which mirrors the way in which we integrate different kinds of knowledge into our own mental models or understanding.

System dynamics, as with any operational modeling, is time consuming and requires specialized training (Valcourt et al., 2020). Simulation modeling also requires either time series data or some other basis for determining parameter values, which can limit its utility. Moreover, because variables are modeled as aggregate quantities, the system dynamics approach excludes network effects or interactions between individual agents (Aerts et al., 2014). This limitation is significant for TBI, which is highly sensitive to localized network dynamics related to brain topography. Therefore, system dynamics models that attempt to capture acute recovery mechanisms should be used alongside approaches that account for highly-variable network within-subject variabilities and between-subject heterogeneities. For this reason, the systems dynamics models that succeed are those able to narrow spatiotemporal questions related to the time course of specific mechanisms and avoid generalizing individual mechanistic explanations from the aggregate patterns observed.

Of the range of applications detailed in this mini-review, the use of causal-loop diagramming to integrate findings from different knowledge and information sources is perhaps the most unique contribution. Causal-loop diagrams can be particularly relevant to understanding the counterintuitive outcomes often observed among survivors of TBI, after controlling for objective variables such as severity of injury (Hawkins et al., 1996; Rath et al., 2003; O’Donnell et al., 2005; Murphy et al., 2006). For example, McLean et al. (2014) found that while higher levels of social activity are associated with higher levels of happiness, increased social activities for TBI patients was insufficient to improve reported quality of life. Authors suggested that patients’ subjective experience of their social activities could be a mediating factor influencing the observed outcome. Systems modeling could serve to reveal the underlying processes generating this and other counterintuitive outcomes.

Future TBI research could benefit from group model building to engage survivors, family and caregivers, and other stakeholders in the process of visualizing the recovery process from the “as-lived” perspective (Hovmand 2014; Siokou et al., 2014; Condon 2019). From there, a simulation model could be co-constructed. Concurrent collection of large-scale observational data could facilitate population of the model with time course data, to generate TBI recovery trajectories. When adequately supported with empirical data, simulation modeling may be able to help improve individualized treatment and care delivery. Further use of causal-loop diagramming and simulation modeling to aid in implementation of evidence-based guidelines is also a promising application.
